# Population-Based Serologic Survey of *Vibrio cholerae* Antibody Titers before Cholera Outbreak, Haiti, 2022

**DOI:** 10.3201/eid2909.230174

**Published:** 2023-09

**Authors:** Christy H. Clutter, Molly B. Klarman, Youseline Cajusma, Emilee T. Cato, Md. Abu Sayeed, Lindsey Brinkley, Owen Jensen, Chantale Baril, V. Madsen Beau De Rochars, Andrew S. Azman, Maureen T. Long, Derek Cummings, Daniel T. Leung, Eric J. Nelson

**Affiliations:** University of Utah, Salt Lake City, Utah, USA (C.H. Clutter, O. Jensen, D.T. Leung);; University of Florida, Gainesville, Florida, USA (M.B. Klarman, Y. Cajusma, E.T. Cato, M.A. Sayeed, L. Brinkley, V.M. Beau De Rochars, M.T. Long, D. Cummings, E.J. Nelson);; Université d'État d'Haïti, Port au Prince, Haiti (C. Baril);; Johns Hopkins University, Baltimore, Maryland, USA (A.S. Azman)

**Keywords:** cholera, Vibrio cholerae, bacteria, antibodies, enteric infections, outbreaks, serosurveys, vibriocidal, correlates of protection, dried blood spots, Haiti

## Abstract

A *Vibrio cholerae* O1 outbreak emerged in Haiti in October 2022 after years of cholera absence. In samples from a 2021 serosurvey, we found lower circulating antibodies against *V. cholerae* lipopolysaccharide in children <5 years of age and no vibriocidal antibodies, suggesting high susceptibility to cholera, especially among young children.

In October 2010, a United Nations peacekeeping mission to Haiti following a highly destructive earthquake inadvertently introduced cholera (*1*,*2*), leading to ≈820,000 cases and ≈10,000 deaths over the following 9 years (*3*). The last confirmed case from that outbreak was reported in January 2019 (*4*), commencing a 3-year period with no confirmed cases. Unfortunately, following a wave of sociopolitical instability that compromised sanitation, 2 cases of cholera were reported on October 2, 2022, and a new outbreak began thereafter; as of February 24, 2023, the outbreak had led to >33,000 suspected cases and 590 registered deaths (*5*). Phylogenetic analyses suggested the current strain descended from the *Vibrio cholerae* O1 Ogawa strain responsible for the original outbreak (*6*; C.N. Mavian et al., unpub data; http://medrxiv.org/lookup/doi/10.1101/2022.11.21.22282526). Although previous infection or vaccination can provide protective immunity, persons not exposed to cholera during the earlier outbreak would be immunologically naive and at higher risk for infection, a hypothesis supported by high reported rates of cholera in young children (*5*). 

After exposure to *V. cholerae*, the predominant adaptive antibody response is to cholera toxin and lipopolysaccharide (LPS) (*7*). However, the most clearly defined nonmechanistic correlate of protection for cholera is presence of vibriocidal antibodies that target the O-specific antigen of the *V. cholerae* LPS (*8*,*9*). Circulating antibody titers peak within several weeks after infection and slowly wane to baseline over ensuing months, with high levels of variability among patients (*8*). Killed whole-cell oral cholera vaccines (OCVs), such as those distributed during vaccination campaigns in Haiti, are 58% effective for the first 2 years, but effectiveness declines to 26% by 4 years after vaccination (*10*). Children >5 years of age show ≈50% OCV protection level at 2-year follow-up compared with adults (*11*). Because no natural infections were reported and vaccinations were not administered during the 3 years preceding the 2022 outbreak, we investigated the presence of *V. cholerae–*specific antibodies in adults and children by analyzing samples collected in a cross-sectional serologic survey in 2 communes in the Ouest Department of Haiti conducted before the 2022 outbreak. 

## The Study 

We collected dried blood spots from 861 enrolled participants, 564 adults and 297 children (<18 years of age) (Table); 62.6% were female and 37.4% male. A small percentage of participants self-reported previous cholera vaccination (1.2%; n = 10) or clinical disease (4.3%; n = 37). We performed ELISAs on all dried blood spot eluates to assess the quantity of circulating cholera toxin B (CtxB) or *V. cholerae*–specific LPS antibodies. For persons with IgG titers for either epitope >2 SD above the mean, we performed vibriocidal assays to assess presence of functional antibodies (Appendix). 

**Table:** Characteristics of 861 participants from serologic study of *Vibrio cholerae–*specific antibodies before a cholera outbreak in Haiti, 2022

Characteristic	No. (%)
Sex	
M	322 (37.4)
F	539 (62.6)
Age group, y	
Adults ≥18	564 (65.5)
Children 5–17	297 (34.5)
Children <5	112 (13)
Cholera vaccination status	
Vaccinated	10 (1.2)
Not vaccinated	847 (98.4)
Unsure	4 (0.4)
Prior infection	
Yes	37 (4.3)
No	822 (95.5)
Unsure	2 (0.2)

**Figure 1:**
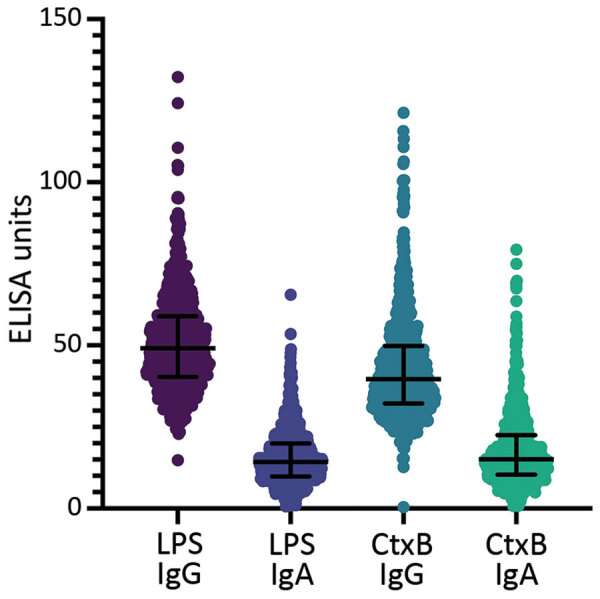
*Vibrio cholerae*–specific and functional antibodies among participants in a serologic study conducted before a cholera outbreak in Haiti, 2022. We performed ELISAs for both IgG and IgA serotypes on all 861 samples for LPS and CtxB. Horizontal lines indicate medians; error bars indicate interquartile ranges. CtxB, cholera toxin subunit B; LPS, lipopolysaccharide.

**Figure 2:**
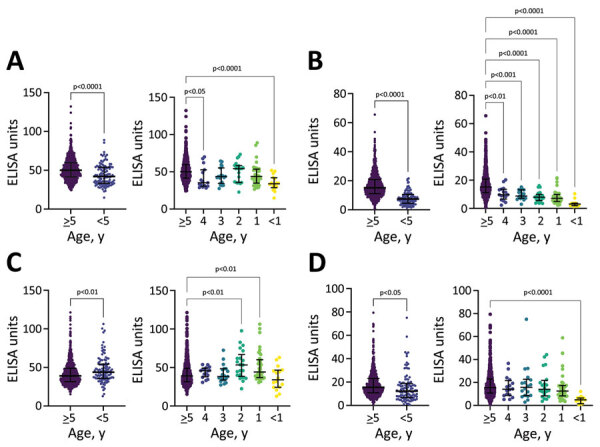
Antibody titers by age, vaccination status, and previous history of infection among participants in a serologic study conducted before a cholera outbreak in Haiti, 2022. We compared antibody titers for lipopolysaccharide (LPS) and cholera toxin subunit B (CtxB) between children <5 years of age (n = 112) and adults and children >5 years of age for LPS IgG (A), LPS IgA (B), CtxB IgG (C), and CtxB IgA (D). We made statistical comparisons between the <5- and ≥5-year age groups using an unpaired 2-tailed Student t test. Individual year-by-year comparisons were compared using 1-way analysis of variance. Horizontal lines indicate medians; error bars indicate interquartile ranges. Significant p values are indicated.

We measured antibody titers for *V. cholerae* LPS and CtxB for both IgG and IgA isotypes in all participants (Figure 1, panel A). Children <5 years of age had significantly lower titers of both LPS IgG and IgA compared with older children and adults (p<0.0001; Figure 2, panels A, B; Appendix Table 1). CtxB IgG was elevated in children <5 years of age (p = 0.0033), especially those 1 (p = 0.0024) or 2 (p = 0.0011) years of age (Figure 2, panel C). We found significant differences in CtxB IgA among children <5 years of age, older children, and adults, but this finding was driven by results from children <1 year of age, who may lack antibodies for reasons unrelated to *V. cholerae* exposure (Figure 2, panel D; Appendix Table 1) (*12*). Using generalized additive model–based splines, we estimated a significant positive nonlinear association of IgA isotypes with age (LPS: effective degrees of freedom [EDF] 3.4, p<0.0001; CtxB: EDF 2.0, p<0.0001) (Appendix Figure 1). This association was not significant for IgG isotypes (LPS: EDF 4.9, p = 0.12; CtxB: EDF 1.0, p = 0.13). We conducted vibriocidal assays on a subset (n = 51/861, 5.9%) of samples (Appendix), but no tested samples had detectable vibriocidal responses (Figure 1, panel B). 

## Conclusions

In this cross-sectional serologic survey within Haiti, we detected low rates of circulating IgG and IgA for LPS and CtxB. Children 1–4 years of age had lower titers of LPS IgG and IgA compared with adults and children >5 years of age. Children 1–2 years of age had elevated CtxB IgG titers, which may reflect the cross-reactive nature of CtxB antibodies with the heat-labile toxin of enterotoxigenic *Escherichia coli*, which has the highest force of infection among enteric pathogens among children in Haiti (*13*). Because of that inherent cross-reactivity for CtxB, LPS IgG is a more specific measure for history of exposure to *V. cholerae,* and the IgG isotype is a more meaningful for comparisons among age groups. However, we detected no vibriocidal antibodies, the best available correlate for protection against cholera. 

Association of results of serologic assays used in our study with previous *V. cholerae* O1 infection has been shown based on longitudinal studies of culture-confirmed cholera patients (*9*) and with protection against disease based on studies of household contacts of index cases and in human challenge studies (*8*,*13*,*14*). Our data were consistent with data on limited recent disease transmission and antigenic exposure in Haiti, especially among young children born during the period in which little pandemic *V. cholerae *was circulating. Those serologic data suggest that persons in communities in Haiti who were serosurveyed, especially children <5 years of age, may have limited preexisting immunologic protection against cholera. 

The 2022 outbreak was caused by a *V. cholerae* Ogawa isolate that aligns with isolates circulating during the 2010–2019 outbreak (*6*; C.N. Mavian et al., unpub data). The degree to which *V. cholerae* circulated in human and environmental reservoirs at a level below the threshold detectable by the surveillance infrastructure during the period between outbreaks is unknown. The intersection between low levels of circulating cholera and declining population immunity, combined with the collapse of clean water and sanitation infrastructure, likely put residents of Haiti at risk for cholera and led to the 2022 outbreak. 

This study was limited by risk of enrollment bias because only 28% of the households screened consented to participate. Given disproportionate sampling in low population density grid cells, true distribution of cholera incidence across the population of Haiti would need to be adjusted before using these data for future serosurveillance research. In addition, observations of relatively lower IgA titers in children 1–4 years of age might have been part of a larger trend of IgA responses increasing with age, a confounding factor that might inaccurately reflect the number of specific exposures. Third, ELISA was limited by availability of quantitative and matrix-matched controls, leading us to use convalescent plasma as positive and naive serum as negative controls. Fourth, selecting samples with high ELISA units for vibriocidal assays may have missed samples with lower antibody levels that harbored functional antibodies. Finally, our serosurvey was limited to 2 adjacent communes in the Ouest Department of Haiti; hence, findings may not be generalizable to other parts of the country. 

In summary, our population-based serosurvey of 2 Haitian communities revealed a lack of functional antibodies and significantly lower *V. cholerae* LPS–specific IgG among young children than older children and adults. These findings suggest persons, especially young children, in Haiti may have high susceptibility to cholera cases and outbreaks. 

Appendix Additional information about study of *Vibrio cholerae–*specific antibodies before a cholera outbreak in Haiti, 2022.
