# Supplementary material for: Population-Based Serologic Survey of Vibrio cholerae Antibody Titers before Cholera Outbreak, Haiti, 2022
Source: Emerg Infect Dis. 2023 Sep;29(9):1864–7. doi: 10.3201/eid2909.230174 (PMC10461687; doi:10.3201/eid2909.230174)
Supplement: Appendix — Additional information about study of Vibrio cholerae–specific antibodies before a cholera outbreak in Haiti, 2022. [file 23-0174-Techapp-s1.pdf]

Article DOI: https://doi.org/10.3201/eid2909.230174

EID cannot ensure accessibility for supplementary materials supplied by authors. Readers who have difficulty accessing supplementary content should contact the authors for assistance.

Population-Based Serologic Survey of *Vibrio cholerae* Antibody Titers before Cholera Outbreak, Haiti, 2022

Appendix

Methods

Ethics statement. The study was reviewed and approved by the Comité National de Bioéthique (National Bioethics Committee of Haiti; 2021-12), the Institutional Review Boards at University of Florida (IRB202002290), and the University of Utah (IRB_00111137).

Study design. Cross-sectional serological survey.

Study population and setting. This study was conducted from February 24th to August 25th, 2021, in the communes of Gressier and Leogane, in the Ouest Department of Haiti. This semi-urban and rural region experienced a cumulative attack rate of 5.1-7.5% early in the original cholera outbreak (2010-2012). Cases decreased substantially over time so that towards the end of the outbreak the entirety of the Ouest Department reported 841 cases in 2018 (*1*). This region was not covered in mass OCV campaigns (*2–5*).

Sampling and randomization. Households were randomly sampled geographically and proportionally to population density across the 477 sq km study area. A sampling grid was constructed inside the study area with 149 total grid cells using ArcGIS (ESRI). Grid cells were stratified by population density (low 0-399, medium 400-1511, high 1512-4664) and the sample schema was established *a priori*. Twenty-five grid cells were randomly chosen proportional to population density (2 low, 5 medium, 18 high). An additional 6 low density grid cells were added to have statistical power to conduct future stratified analyses on population density effects. Logistical constraints resulted in 25 grid-cells sampled (6 low, 3 medium, 15 high) out of the 31 intended to be sampled. Within each grid cell sampled, 21 points were randomly distributed and

enclosed by Thiessen polygons. Within each polygon, one household was enrolled and sampled; the intention was to enroll from 18 of the 21 polygons per selected grid cell; the remaining 3 were used as ‘back-up’ polygons. The order that grid cells were sampled was randomized.

Participant recruitment. Haitian staff navigated to each grid cell and polygon (via car, motorcycle, or foot) using ArcGIS Collector (ESRI). The first household reached upon entering the targeted polygon was screened. If the household declined participation or was ineligible, screening continued by proceeding to the next closest house, while remaining within the polygon. A ‘spin the bottle’ app was used to determine the ‘closest’ house if there were multiple equidistant neighboring houses. This process was repeated until either one household was enrolled per polygon (18 total per grid cell) or all households within the polygon were screened.

Participant inclusion criteria, consent process, and incentives. Eligibility criteria included a household with one adult head of household (HoH) who provided written consent to complete a household survey and at least two household members who provided written consent to provide a dried blood spot (DBS) sample and respond to a household member survey. Parents/guardians 18 years and older provided consent for their minor children and children ≥ 7 years provided assent. Households with a single member were eligible if both consent for the surveys and DBS sample were provided. There was no lower age limit for eligibility. A 500 Gourde ($4.50 US) phone credit was provided to HoH participants to facilitate contact with the study hotline for questions.

Sample collection. DBS sampling was performed by finger stick, or heel stick in children <1 year, and spotted onto Whatman 903 Protein Saver cards.

Data collection. The HoH survey queried family demographics and socioeconomic status. The household member survey asked about current and past symptoms and history relating to diarrheal syndromes. Vaccination and disease history were self-reported. Children under 5 years were measured for basic parameters of malnutrition (mid-upper arm circumference (MUAC), weight, and height).

DBS Eluates. Using a hole puncher, nine 6mm spots were punched from the blood circles on each DBS card and placed into a microcentrifuge tube containing 600 µL of a 1X PBS-Tween solution. The samples were then placed on a tube rocker at speed 50 rpm overnight at

4°C. The next day the samples were centrifuged at 10,500 x g for 2 minutes. The supernatant was removed and stored at -80°C until ready for further use.

ELISA. Enzyme-linked immunosorbent assays (ELISAs) were performed on all DBS specimens (n=861). Nunc Maxisorp flat-bottom plates (Sigma-Aldrich) were coated overnight with either *V. cholerae* O1 Ogawa-specific LPS (gift of Edward Ryan, Massachusetts General Hospital) or monosialoganglioside (GM_1, Sigma-Aldrich) at 1 µg/mL. We focused on the Ogawa serotype as the legacy strain from the prior outbreak and pathogenic culprit of the present one. Antibodies against Inaba-specific LPS were not assessed. Plates that had been coated with GM_1 were washed in phosphate buffered saline (PBS) with 0.05% Tween20 (PBST), blocked in PBST + 1% bovine serum albumin (BSA), and coated for a second night in cholera toxin subunit B (CtxB, Sigma-Aldrich) at 2.5 µg/mL. Following coating, plates were blocked, incubated with DBS eluates that were diluted 1:7.5 in PBST + 0.1% BSA, incubated in 1:1000 horseradish peroxidase (HRP) conjugated secondary antibody, either goat-anti-human IgG or IgA (Jackson ImmunoResearch), and developed in TMB substrate solution (Thermo Scientific). Immediately after addition of TMB, plates were placed on a BioTek ELx800 plate reader and read kinetically at 405nm once every minute for eight minutes. Maximum slope (MaxV) values were normalized against a positive control for each plate to generate ELISA units. Positive controls were convalescent plasma from confirmed cholera cases, diluted 1:100. Negative controls were naïve serum (Sigma-Aldrich), also diluted 1:100.

Vibriocidal antibody assay. The criteria to select samples for vibriocidal antibody assays were those samples with an IgG ELISA titer two standard deviations above the mean for either LPS IgG or CtxB IgG. The assay was conducted via a previously described DBS eluate-adapted drop-plate vibriocidal method (5) and adapted for this study. *V. cholerae* O1 El Tor Ogawa strain (X25049) were grown overnight on thiosulfate-citrate-bile salts-sucrose (TCBS) *Vibrio*-selective agar, after which 2-3 colonies were cultured in Luria-Bertani (LB) broth, washed several times with sterile PBS and adjusted to an OD_{600} of 0.3. DBS samples were diluted 1:5 in sterile PBS, heat-inactivated at 56°C, and then serially diluted 1:2 horizontally across a flat-bottom Nunclon™ Delta Surface 96-well plate (Thermo Scientific). Each 96-well-plate included three growth controls and three negative controls. Each batch included at least one monoclonal antibody positive control. Samples and positive controls were cultured for 1hr in a growth solution containing the normalized *V. cholerae* culture (OD_{600} = 0.3) and guinea pig

complement (Sigma-Aldrich). Negative controls were cultured with sterile PBS. Plate controls were subsequently plated in 5µL drops onto LB agar. Positive control and participant samples were plated onto TCBS agar (selective media for *V. cholerae*) and cultured overnight at 37°C. Titers were determined as the reciprocal of the first dilution in which the culture drop shows serrated edges or individual colonies.

Statistical Analyses. Statistical comparisons between two populations were assessed with an unpaired, two-tailed Student's t test. The significance threshold was set at $\alpha = 0.05$. Generalized additive models (GAMs) with a spline to fit age were used to assess the relationship of age and antibody distribution. R version 4.1.3 with the mgcv package was used to fit the splines.

References

1. United Nations Office for the Coordination of Humanitarian Affairs. Haiti Cholera figures; as of 31 January 2019. 2019 [cited 2023 Feb 7]. https://www.humanitarianresponse.info/sites/www.humanitarianresponse.info/files/documents/files/ocha-hti-cholera-figures-20190131_en.pdf

2. Ivers LC, Teng JE, Lascher J, Raymond M, Weigel J, Victor N, et al. Use of oral cholera vaccine in Haiti: a rural demonstration project. Am J Trop Med Hyg. 2013;89:617–24. PubMed https://doi.org/10.4269/ajtmh.13-0183

3. Rouzier V, Severe K, Juste MAJ, Peck M, Perodin C, Severe P, et al. Cholera vaccination in urban Haiti. Am J Trop Med Hyg. 2013;89:671–81. PubMed https://doi.org/10.4269/ajtmh.13-0171

4. Pan American Health Organization. Haiti's Ministry of Health organizing a vaccination campaign against cholera in areas affected by Hurricane Matthew, supported by PAHO-WHO, UNICEF and other partners. 2016 [cited 2023 Feb 7]. https://www3.paho.org/hq/index.php?option=com_content&view=article&id=12682

5. Iyer AS, Azman AS, Bouhenia M, Deng LO, Anderson CP, Graves M, et al. Dried blood spots for measuring *Vibrio cholerae*–specific immune responses. PLoS Negl Trop Dis. 2018;12:e0006196.

Appendix Table. Descriptive Statistics for Age Comparisons*

Category	**Median**	**Range**	**25th Percentile**	**75th Percentile**	**p value**
LPS IgG					
≥5 years old	50.00	107.9	41.62	59.87	
<5 years old	43.73	66.11	34.60	54.88	**<0.0001**
4 years old	35.36	41.87	30.66	52.66	**0.0341**
3 years old	43.73	36.82	34.49	55.23	0.1018
2 years old	54.14	50.58	35.73	58.31	0.9633
1 year old	43.77	62.41	34.80	53.59	0.0595
<1 year old	33.96	37.12	28.65	42.03	**<0.0001**
LPS IgA					
≥5 years old	15.15	64.89	11.19	20.85	
<5 years old	8.075	19.43	6.038	11.20	**<0.0001**
4 years old	9.675	17.97	6.628	13.52	**0.0052**
3 years old	8.770	12.18	7.140	13.16	**0.0003**
2 years old	7.940	12.59	5.590	9.955	**<0.0001**
1 year old	7.175	19.43	4.930	9.928	**<0.0001**
<1 year old	3.075	9.100	1.953	3.730	**<0.0001**
CtxB IgG					
≥5 years old	39.12	106.0	31.55	49.02	
<5 years old	45.11	83.92	36.78	55.85	**0.0033**
4 years old	45.77	23.11	37.98	49.23	0.9937
3 years old	38.23	45.07	33.87	48.46	0.9993
2 years old	53.30	75.26	38.50	66.82	**0.0024**
1 year old	44.22	75.73	36.96	60.15	**0.0011**
<1 year old	34.01	50.40	24.36	46.60	0.4507
CtxB IgA					
≥5 years old	15.50	78.58	10.81	23.08	
<5 years old	13.95	72.12	8.620	19.77	**0.0138**
4 years old	14.09	31.46	8.738	21.62	0.8780
3 years old	15.75	72.12	8.520	22.76	0.9979
2 years old	14.03	40.64	8.278	21.80	0.9999
1 year old	12.50	55.40	8.265	17.38	0.7348
<1 year old	4.900	11.41	1.440	6.470	**0.0004**

**The median, interquartile range and p-value for each of the age comparisons is shown. Children younger than 5 years were compared in aggregate to children and adults 5 years old and older using an unpaired two-tailed student t test. Individual age groups of 1, 2, 3, and 4-years were compared to older children and adults by one-way ANOVA. Units for all columns excluding the p-value refer to ELISA units. Bold indicates significant values.*

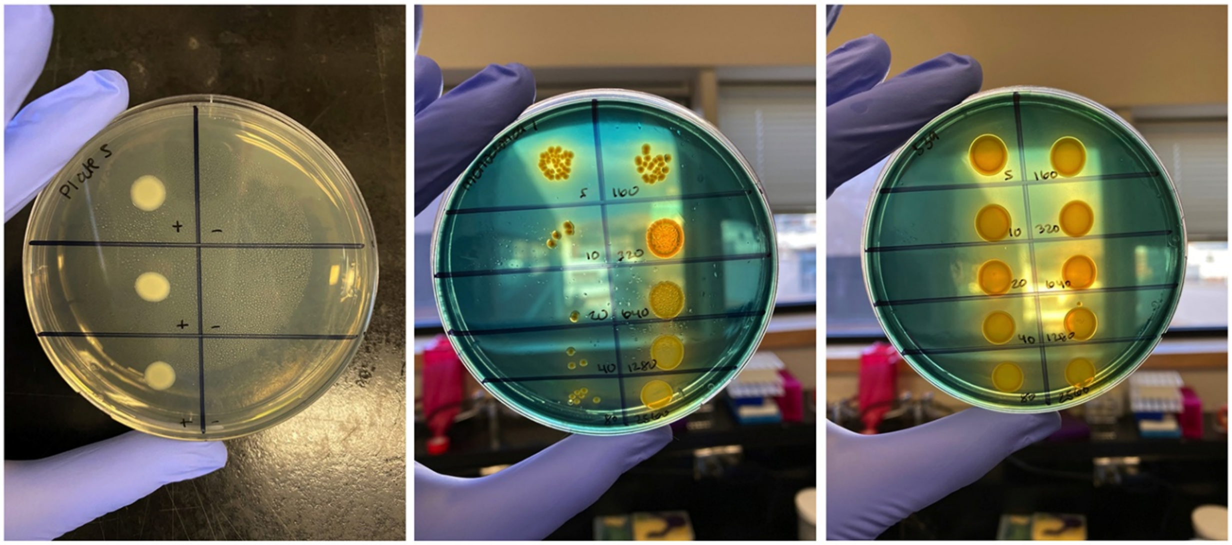

Appendix Figure 1. Representative vibriocidal drop plates. Samples that were at least 2 SD above mean for LPS or CtxB IgG (n = 51) were assayed for functional (vibriocidal) antibodies using a drop plate serial dilution method. Representative plates are pictured. LPS, lipopolysaccharide; CtxB, cholera toxin subunit B.

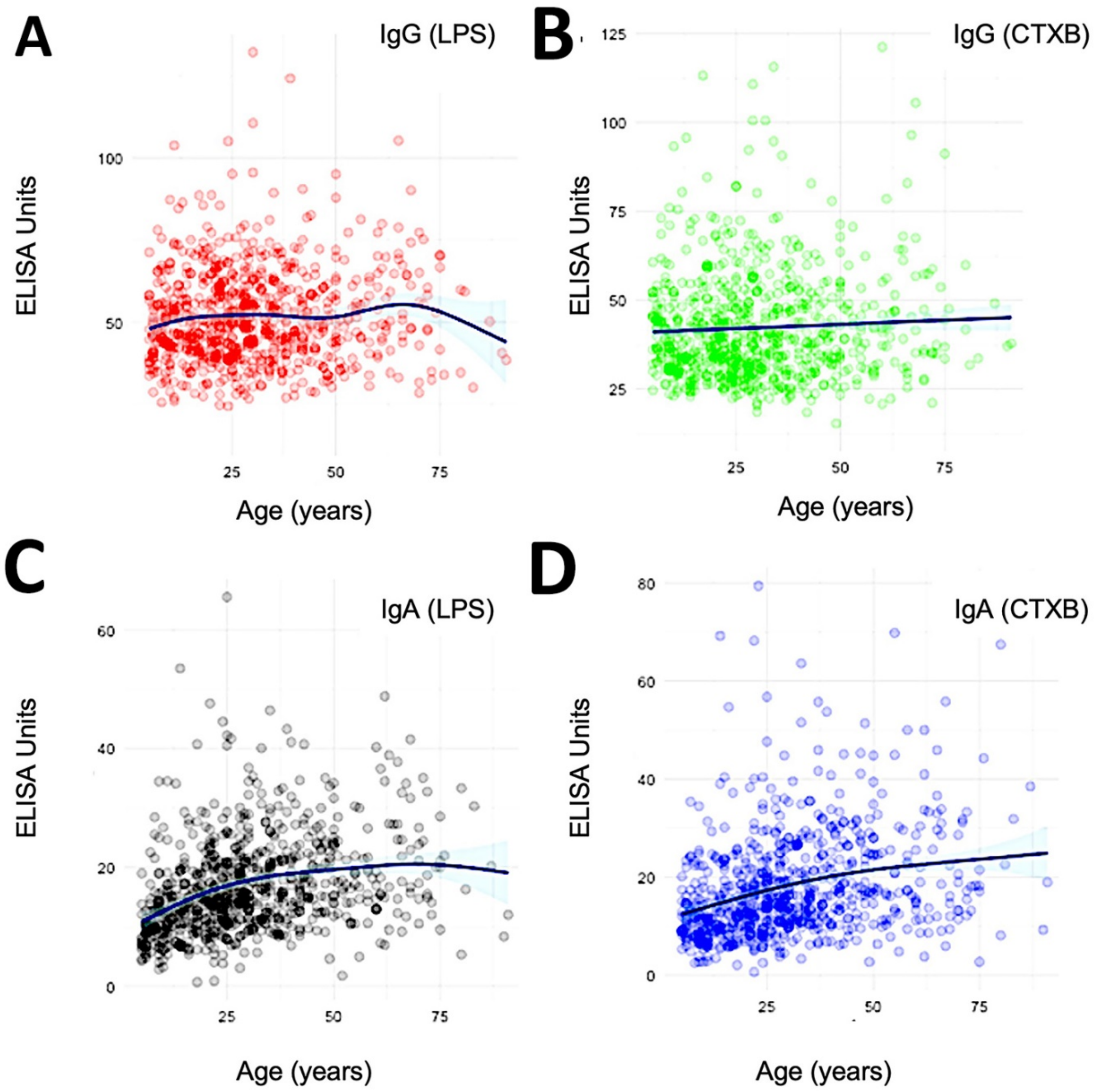

Appendix Figure 2. Antibody levels expressed in ELISA units analyzed by age of participant using a generalized additive model (GAM). A) IgG to *Vibrio cholerae* LPS (effective degrees of freedom [EDF] 4.9, p = 0.12); B) IgG to *V. cholerae* cholera toxin subunit B (CtxB; EDF 1.0, p = 0.13); C) IgA to *V. cholerae* LPS (EDF, 3.4, p<2e-16); D) IgA to *V. cholerae* CtxB (EDF 2.0, p<2e-16). A statistically significant association (p<0.05) was identified between age and IgA for both LPS and CtxB.
